# Identifying Protein-Protein Interaction Sites Using Covering Algorithm

**DOI:** 10.3390/ijms10052190

**Published:** 2009-05-15

**Authors:** Xiuquan Du, Jiaxing Cheng, Jie Song

**Affiliations:** The Key Laboratory of Intelligent Computing and Signal Processing, Ministry of Education, Anhui University, Anhui, China; E-Mails: cjx@ahu.edu.cn (J.-X.C.); jsong@ahu.edu.cn (J.S.)

**Keywords:** protein-protein interaction, covering algorithm, sequence profile, residue accessible area, maximum entropy

## Abstract

Identification of protein-protein interface residues is crucial for structural biology. This paper proposes a covering algorithm for predicting protein-protein interface residues with features including protein sequence profile and residue accessible area. This method adequately utilizes the characters of a covering algorithm which have simple, lower complexity and high accuracy for high dimension data. The covering algorithm can achieve a comparable performance (69.62%, Complete dataset; 60.86%, Trim dataset with overall accuracy) to a support vector machine and maximum entropy on our dataset, a correlation coefficient (CC) of 0.2893, 58.83% specificity, 56.12% sensitivity on the Complete dataset and 0.2144 (CC), 53.34% (specificity), 65.59% (sensitivity) on the Trim dataset in identifying interface residues by 5-fold cross-validation on 61 protein chains. This result indicates that the covering algorithm is a powerful and robust protein-protein interaction site prediction method that can guide biologists to make specific experiments on proteins. Examination of the predictions in the context of the 3-dimensional structures of proteins demonstrates the effectiveness of this method.

## 1. Introduction

Protein-protein interactions and protein-DNA interactions are among the most ubiquitous types of macromolecule interactions in biological systems. Revealing the mechanisms of protein-protein interactions is crucial for understanding the functions of biological systems. Furthermore, the ability to predict interfacial sites is also important in mutant and drug design [1]. Structural knowledge at the residue and atom level is one of the keys to understanding the mechanisms of protein interactions. X-ray crystallography and NMR are without doubt the best methods to obtain such information. In recent years, high throughput technologies have provided experimental tools to identify protein-protein interactions systematically and have generated tremendous amount of protein interaction data. However, the high throughput experiments are often associated with high numbers of false positives and false negatives [2]. The experiments are also tedious and labor-intensive and they cannot meet the requirements of proteomics, since there can be many thousands of protein-protein interactions even for a relatively primitive organism, so the need arises for seeking complementary *in silico* methods that are capable of accurately predicting interactions.

The availability of more and more protein structures in the Protein Data Bank (PDB) [3] makes prediction of protein-protein interaction sites possible. A series of computational efforts to identify interaction sites or interfaces in proteins have been undertaken, such as hydrophobic residues cluster at some interfaces [4,5], Jones and Thornton have proposed two kinds of complexes: ‘permanent’ and ‘transient’ [6] and so on. Current biophysical theories about the protein interacting regions highlight the role of the shape, chemical complementarily and flexibility of the molecules involved [7]. In parallel, a growing number of machine learning methods for inferring protein interactions have been proposed, such as neural networks (ANN) [8–10] and support vector machines (SVMs) [11–15] have been successfully applied in this field. These studies consider sequential, structural or evolutionary features such as amino acid residue composition [8,10,13,14,16], spatial neighboring residues [15,16], accessible surface area, structural conservation score and residue evolutionary information. However, Res I. *et al.* [14] use protein sequential and evolutionary information to predict protein interaction sites without structural information.

Traditional methods take protein-protein interaction site prediction as a classification task and separately study each residue. Li Ming-Hui *et al.* [17] take it as a sequence labeling task using conditional random fields (CRFs) in their research.

In this study, we mainly focus on a novel method developed for detecting interacting surfaces in proteins starting from their three-dimensional structure. This is particularly important in determining protein function, particularly for proteins of known structure but unknown function. Ofran *et al.* [18] investigated the sequence neighborhood of protein-protein interface residues in a set of 333 proteins and found that 98% of these interface residues have at least one additional interface residue within their local sequence vicinity, so we think the characteristic that protein interface residues tend to form spatial clusters can be an important factor in solving our problem. A new method is constructed to learn association rules at the protein surface, i.e. a covering algorithm system. We also discuss the prediction power of support vector machines (SVMs), the covering algorithm (CA) and maximum entropy (ME) [19].

## 2. Results and Discussion

### 2.1. Cross-validation

The covering algorithm method is trained to predict whether or not a surface residue which is located in the interface based on identity of the target residue and its sequence neighbors. Five-fold cross-validation strategy is adopted for our experiments. Specifically, on the each dataset, we divide our dataset to five parts according to 5-fold cross-validation. The training set is composed of four parts and the remainder is the testing set. Thus, we get five training sets and testing sets. Then, we carry out our experiment on these five training sets and testing sets. For each dataset (see collection of dataset), we do ten times. Herein, total 2 × 5 × 10 = 100 experiments are implemented and the average performance of the results is used to evaluate each method.

### 2.2. Evaluation measures of the covering algorithm (CA)

The covering algorithm (CA) classifier is evaluated using 5-fold cross-validation on two kinds of datasets. Table 1 shows the classification performance as measured by correlation coefficient, accuracy, specificity, sensitivity and F1-measure. Of the residues predicted to be interface, 58.83% (Complete), 53.34% (Trim) are actually interface residues, and 56.12% (Complete), 65.59% (Trim) of interface residues are identified as such. We also investigate the fraction of interface residues in each protein that are correctly identified by the covering algorithm (CA) classifier. In eight out of 12 (~ 75%) proteins the classifier can recognize the interaction surface by identifying at least half of the interface residues. and in 92% of the proteins, at least 40% of the interface residues are correctly identified.

In order to examine whether the covering algorithm (CA) method learns sequence characteristics that are predictive of target residue functions, we run a control experiment in which the class labels are randomly shuffled. The correlation coefficient (CC) obtained on the class shuffled dataset is 0.0604 (our method with 0.2893 on the Complete data) and −0.0065 (our method with 0.2124 on the Trim data) shows that the covering algorithm performs better than a random predictor (CC ≈ 0). Table 1 shows the result between the covering algorithm and random classifier. From this table, the covering algorithm has got better performance (5% ~ 10% sensitivity, 7% ~ 11% specificity, 10% ~ 14% accuracy, 13% ~ 14% F1-measure and 21% ~ 23% CC, respectively) than a random classifier.

### 2.3. FP rate versus TP rate tradeoff

**Figure 1.**
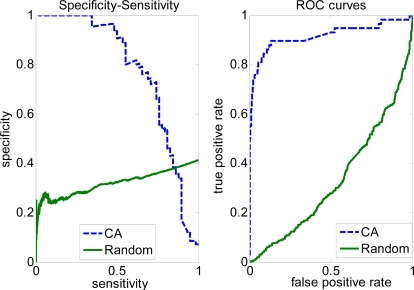
Specificity-sensitivity and ROC curves on the Complete dataset.

**Figure 2.**
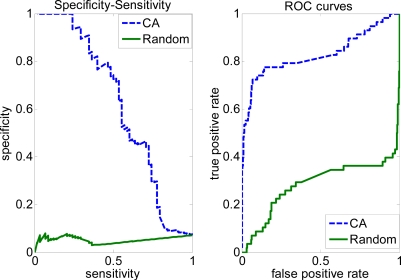
Specificity-sensitivity and ROC curves on the Trim dataset.

In some situations (e.g. key interface residue recognition for site-specific mutagenesis), we need to have a higher sensitivity and lower specificity. This requirement can be met by modifying the parameters used by the covering algorithm (CA). Figure 1 shows the specificity-sensitivity graph and ROC curves for the Complete dataset. Figure 2 shows specificity-sensitivity graph and ROC curves for the Trim dataset. The area under the ROC curve (AUC = 0.9167) of the covering algorithm for the Complete dataset is higher than the random classifier with 0.3307 (random), and AUC (0.8298) from the covering algorithm (CA) of the Trim dataset is larger than random classifier with 0.2847 (random). But AUC decreases about 8% using the covering algorithm between the Complete and Trim dataset, this perhaps because of removing some non-interfacial residues from training set (Complete dataset) reduce the performance of the covering algorithm method and these removed residues may contain useful information for predicting interaction sites.

### 2.4. Comparison with other methods

Support vector machines (SVMs) and maximum entropy model (ME) are selected to compare with our method. They are all discriminative classification methods. SVMs are a state-of-art method for predicting protein-protein interaction sites [11,13,15,16,28]. ME is implemented in [17]. Herein, we evaluate these methods using 5-fold cross-validation on the same dataset for direct comparison with our method. LIBSVM is used as the SVM implementation with radial basis function as kernel and default C, γ. Stanford Classifier (ME) is used and can be download freely from http://www-nlp.stanford.edu/software/classifier.shtml.

Table 2 shows the results using covering algorithm (CA), support vector machine (SVM) and maximum entropy (ME) on the Trim and Complete dataset. From the Table, we find that our classifier has good performance in our dataset. The covering algorithm (CA) performs best, according to sensitivity, F1-measure, accuracy and CC, but its specificity was slightly lower than that of SVM and ME on the Complete dataset. In the Trim data, the sensitivity, F1-measure and CC achieved by the CA method is higher than SVM (7.52% better sensitivity and 2.27% better F1-mearsure and 0.92% better CC), albeit with 5.47% lower specificity. If judged only by sensitivity, the CA seems to slightly outperform (by 4%) the ME, whatever the dataset. Experiments on our dataset shows that CA is an effective method for protein interaction sites recognition, especially for Complete dataset.

**Figure 3.**
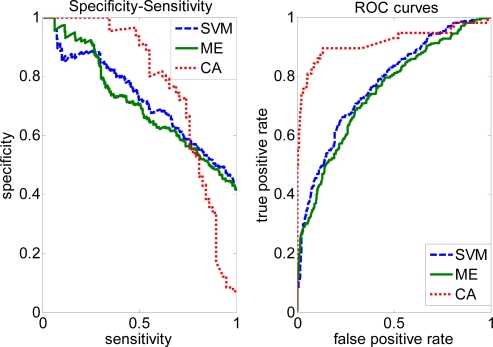
Specificity-sensitivity and ROC curves on the Complete data based on SVM, ME and CA.

In order to illustrate the effectiveness of our approach, we plotted the ROC curves for the Complete and Trim datasets. As shown in Figure 3, prediction performance is improved by the CA method with higher AUC = 0.9167 than SVM (0.7754), ME (0.7486) on the Complete dataset. After removing some negative samples (i.e. Trim dataset), performance of the CA method (0.8298) is slightly lower, but still larger than SVM (0.7654) and ME (0.7488).

### 2.5. Some experimental examples

**Figure 5.**
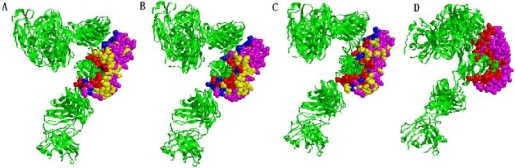
Predicted interface residues (red color) on protein (PDB: 1NFD_C) identified by (A) CA, (B) SVM (C) ME and (D) The actual interface residues. Red denotes true positive residues, blue denotes false negative residues, yellow denotes false positive residues, and pink denotes true negative residues.

Here we give two examples that are predicted by the CA, SVM and ME classifiers. The first example is the refined 2.8 an alphabeta T cell receptor (TCR) heterodimer complexed with an anti-TCR fab fragment derived from a mitogenic antibody [21]. We use our classifier to predict 45 residues to be interfaced with 81.82% sensitivity and 55.56% specificity (Figure 5A). SVM predicts 38 interface residues with 69.09% sensitivity, 58.46% specificity (Figure 5B). ME predicts 34 interface residues with 61.81% sensitivity, 52.30% specificity (Figure 5C) while the actual interface residues are 55 (Figure 5D).

**Figure 6.**
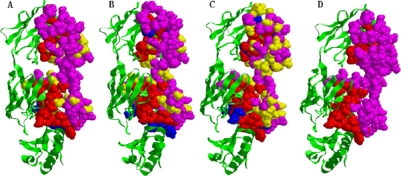
Predicted interface residues (red color) on protein (PDB: 2JEL_H) identified by (A) CA, (B) SVM (C) ME and (D) The actual interface residues. Red denotes true positive residues, blue denotes false negative residues, yellow denotes false positive residues, and pink denotes true negative residues.

The second example is the jel42 Fab fragment/HPr complex [22]. This interface region is accurately identified by CA covering ~ 83% of the actual binding site with a specificity of 63.93% (Figure 6A), The prediction result by SVM covers only 78.26% of the actual binding site with a specificity of 62.06% (Figure 6B). ME predicts 34 interface residues with 73.91% sensitivity, 37.78% specificity (Figure 6C) versus the number of actual interface residues which are 46 (Figure 6D).

## 3. Experimental

Each surface residue is predicted to belong to a particular interaction site on the basis of characteristic of residue spatial cluster. Interaction site residues and non-interaction residues are used as positive and negative data, respectively.

### 3.1. Collection of data sets

In our experiments protein-protein interaction data are extracted from a set of 70 protein-protein complexes in an independent study [20] that contain X-ray diffraction structures of protein-protein complexes determined at a resolution of 1.6 Å or better. The dataset eliminates homo-complexes whose interacting surfaces are characterized by hydrophobicity. In order to obtain non-redundant protein chains of hetero-complexes we adopt two processes. First, all chains of 70 protein complexes are compared assigned using the BLASTCLUST program of NCBI BLAST 2.0. Two chains are assigned with the same cluster if (1) over 90% of their sequences are aligned and (2) the sequence identity is > 30%. All above chains are clustered in this way. The first chain of each cluster is selected. Second, protein chains shorter than 40 residues are removed and we select protein chain pairs with ≥ 20 interfacial residues. A residue is considered to form an interfacial contact if the distance between α-carbon atoms and any α-carbon atoms of its interacting proteins are < 1.2 nm [9]. For protein chains that interacts with multiple partners, only one partner with the most interfacial residues is selected. According to the above definitions, the finally dataset is composed of 61 hetero-complexes, which includes 12 protease-inhibitor complexes, five antibody-antigen complexes, eight enzyme complexes, eight G-proteins, cell cycle, signal transduction and seven miscellaneous complexes. The dataset used is available online as Supplementary Material at *IJMS*.

Interfaces are formed mostly by residues that are exposed to the solvent if the partner chain is removed, so we mainly focus on surface residues. The solvent accessible surface area (ASA) is computed for each residue in the unbound molecule (MASA) and in the complex (CASA) using the DSSP program [23]. Here, we should emphasize that only the coordinates of the unbound chain are used in the calculation. If other chains present in the complex are included, their influence would cause the ASA to be incorrectly calculated. In this paper, a residue is considered to be a surface residue if its relative accessible surface area (ASA) is at least 16% of its nominal maximum area whose value as defined by [24]. As a result, a total of 6,567 residues (~ 64.03%) are collected as surface residues from all these chains. A surface residue is defined to be an interface residue if it formed an interfacial contact. According to this definition, we get about 24.03% (2,465) of all surface residues in the dataset.

The fact that there are more non-interface residues than interface residues in the training set leads to higher specificity and lower sensitivity for many classifiers such as SVMs and ANN [8,13]. In order to evaluate the robustness and performance of different methods, we conduct experiments on Trim dataset and Complete dataset. Table 3 shows Complete and Trim dataset. The entire cross-validation procedure is repeated ten times, and the resulting average performances are used to evaluate our method.

### 3.2. Generation of the character vector

Interface prediction relies on characteristics of residues found in interfaces of protein complexes. The characteristics of interface residues are different. The most prominent involve: sequence conservation, proportions of the 20 types of amino acids, secondary structure, solvent accessibility and side-chain conformational entropy etc. Most of these characters are structure information. In this article, we choose sequence profile and residue accessible surface area as our test character.

#### 3.2.1. Protein sequence profile feature

Sequence profiles are sequence information which denotes its potential structural homolog. Protein function information is embedded in the protein sequence, but how it can be determined is a pivotal problem. A good candidate technique for extracting such information is multiple sequence alignment (MSA). Protein sequence profile is a result of MSA which shows which kind of amino acid appearing in a given position of the protein primary structure. Herein, the protein sequence profiles are extracted from the HSSP database [27]. Each residue is coded as a vector of 20 elements which denotes relative frequency for each of the 20 amino acid residue in a given sequence position, from counting the residue at that position in each of the aligned sequences including the test sequence.

#### 3.2.2. ASA feature

Accessible surface area (ASA) feature represents the relative accessible surface area (scaled by the nominal maximum area of each residue). For convenience, we use ASA to represent the relative accessible surface area of residue. ASA of each residue is calculated using DSSP program [23].

In order to include the environment of the target residue, the profiles of sequentially neighboring residues with n windows are also included in the character vector. Equation (1) is an example of a vector with 11 windows in our experiment:

(1) $${\text{V}}_{\text{n}}\;=({\text{p}}_{\text{n}-5,1},\ldots ,{\text{p}}_{\text{n}-5,20},{\text{p}}_{\text{n}-5,21},{\text{p}}_{\text{n},1},\ldots {\text{p}}_{\text{n},21},\ldots ,{\text{p}}_{\text{n}+5,1},\ldots ,{\text{p}}_{\text{n}+5,20},{\text{p}}_{\text{n}+5,21})$$

and:

$$\{\begin{array}{cc} {\text{p}}_{\text{n},\text{j}}=\frac{{\text{N}}_{\text{n},\text{j}}}{\sum_{\text{j}}{\text{N}}_{\text{n},\text{j}}} & \text{j}=1\ldots 20\\ {\text{p}}_{\text{n},\text{j}}=\text{ASA}({\text{x}}_{\text{n}}) & \text{j}=21 \end{array}$$

where N_n,j_ is the number of amino acids j in position n, X_n_ is a residue in position n and ASA(X_n_) denotes accessible surface area of residue X_n_.

### 3.3. Covering algorithm (CA) for classification

**Figure 7.**
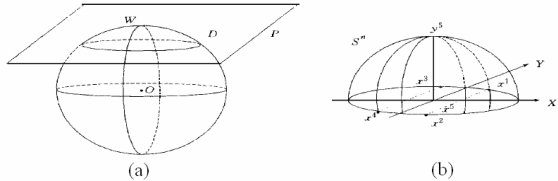
(a) a sphere neighborhood (b) input vector and their projection.

Data-based machine learning explores the rule to predict new data from the observation data. The covering algorithm is proposed by Zhang Ling and Zhang Bo for classification. Suppose that given input set *K* = {*X*_1_, *X*_2_,......*X_K_*} (K is a set of points in the N dimension Euclid Space, *X*_1_ = (*x*_1_, *y*_1_), *X*_2_(*x*_2_, *y*_2_), ......*X_k_* = (*x_k_, y_k_*), *x*_1_, *x*_2_,......, denotes input vector of covering algorithm, *y*_1_, *y*_2_,... *y_k_* ∈ {1, −1} denotes label of *x*_1_, *x*_2_,...xk). Now suppose K is divided to s subsets: in this paper, we discuss s = 2 (i.e. two classes corresponding to interface residue and non-interface residue). First, the original input space (*K*_1_, *K*_2_) is transferred into a quadratic space by the use of a global project function, such as Figure 7. Then, the well-known point set covering method is used to perform the partition of the data in the transformed space.

#### 3.3.1. Algorithm 1

Step 1. Making a cover C(i) (i = 1 at the begin), which only covers point of K_1_ and these points are enclosed set D.

Step 2. Taking point of K/D, i.e. p, suppose p belongs to K_j_(j = 1, 2), making a cover C(i) which only covers point of K_j_, and then are enclosed set D, i = i + 1, return Step 2 until K/D=Φ.

Step 3. Suppose we get cover set C ={C_1_, C_2_,......C_k_}. Then taking C_1_, C_2_,……., C_k_, if test point is in the C_i_ which cover point of K_1_, output 1, otherwise −1.

In fact, C(i) is a sphere domain with center w and radius r_i_.

#### 3.3.2. Algorithm 2 for making a cover C(i)

Step 1. if K_1_ or K_2_ is empty, then stop. Otherwise, suppose that K_1_ ≠ Ø, randomly selecting a_i_ ∈ k_1_(j = 1, i = 1 at the begin).

Step 2. Seeking a sphere domain with center= *a_i_*. Suppose C(a_i_) ∩ K_1_ = D_i_, i = 1,2..., D_0_ = Ø.

(2) $${\text{d}}_{1}(i)=\underset{x\notin k_{1}}{\text{max}}\{<a_{i},\;x>\}$$

(3) $${\text{d}}_{2}(i)=\underset{x\notin k_{1}}{\text{max}}\{<a_{i},\;x>|<a_{i},x>≻d_{1}(i)\}$$

(4) $$\text{d}(i)=\frac{d_{2}(i)+d_{1}(i)}{2}$$

(5) $$\{\begin{array}{c} \theta_{i}=d(i)\\ \omega =(a_{i}) \end{array}$$

Step 3. C_j_ = C(a_i_), K_1,j_ = C_j_ ∩ K_1_, K_2_ ← K_1_ / K_1,j_, k_1_ ← k_2_, j ← j+1, go to Step 1 of Algorithm 1.

More details about covering algorithm can be referred from [25,26].

Hence by using the training set we can calculate all the parameters *W* = {*ω* = (*a_i_*), *θ* = (*θ_i_*)}based on the above equations and by using testing set, the performance of our algorithm can be evaluated.

### 3.4. Predictor construction

In our experiment, predictors are generated using the covering algorithm (CA) to judge whether a residue is located on an interface or not. The CA has simple, lower complexity, high accuracy for high dimension data and frequently demonstrates high accuracy. It can also handle large feature spaces and condense the information given by the training dataset. Here, we consider only surface residues in the training process, the target value of which is 1 (positive sample) if it is classified into interface residue and −1 denotes non-interface residue corresponding to negative sample.

We construct our CA predictor using sequence profile and ASA attributes. Following the method used by Fariselli *et al.* [9], the input vector of CA is fed with a window of 11 residues, centered on the target residue and including the five sequence neighboring residues on each side such as formula (1) organization. So, each residue is represented by a 231-component vector in the predictor based on the residue sequence neighboring profile and ASA.

### 3.5. Evaluation of performance

Interface prediction has to fulfill two competing demands. The predictor should cover as many of the real interface residues as possible, but at the same time should predict as few false positive as possible. These two demands are measured by sensitivity and specificity, respectively. Let TP = the number of true positives (residues predicted to be interface residues that actually are interface residues); FP = the number of false positives (residues predicted to be interface residues that are in fact not interface residues); TN = the number of true negatives; FN=the number of false negatives; N = TP + TN + FP + FN (the total number of examples), then sensitivity is:

$$\text{sensitivity}=\frac{\text{TP}}{\text{TP}+\text{FN}}$$

and specificity is:

$$\text{specificity}=\frac{\text{TP}}{\text{TP}+\text{FP}}$$

and correlation coefficient (CC) is:

$$\text{CC}=\frac{\text{TP}*\text{TN}-\text{FP}*\text{FN}}{\sqrt{\left(\text{TP}+\text{FN})*(\text{TP}+\text{FP})*(\text{TN}+\text{FP})*(\text{TN}+\text{FN}\right)}}$$

and F1-measure is:

$$\text{F}1-\;\text{measure}=\frac{2*\text{sensitivity}*\text{specificity}}{\text{sensitivity}+\text{specificity}}$$

Sensitivity measures the fraction of interface residues that are identified as such. Specificity measures the fraction of the predicted interface residues that are actually interface residues. Correlation coefficient measures that how well the predicted class labels correlate with the actual class labels. It ranges from −1 to 1 where a correlation coefficient of 1 corresponds to perfect prediction and 0 corresponds to random guessing.

## 4. Conclusions

Generally speaking, identifying residues in protein-protein interaction sites is an extremely difficult task, let alone in the absence of any information about partner chains. In this paper, as we have presented above, due to the absence of information about research proteins, we propose a new approach to predict interface sites from protein sequence and structure characteristic. This method adequately utilizes the characters of covering algorithm which have simple, lower complexity, high accuracy for high dimension data. A relatively high false positive ratio in protein-protein interaction sites prediction is a troublesome problem. Some investigators reduce the false positive ratio by eliminating isolated raw positive predictions [13]. In our experiment, we can decrease false positive predictions using a covering algorithm based on different features of protein-protein interaction. The results obtained in this paper show that our propose method is a promising approach for studying protein-protein interaction, although this method is not good in sensitivity. Choosing proper features perhaps improve the results and we will investigate more effective features in the future and information of binding protein chains will also be considered in our future work.

## Figures and Tables

**Figure 4.**
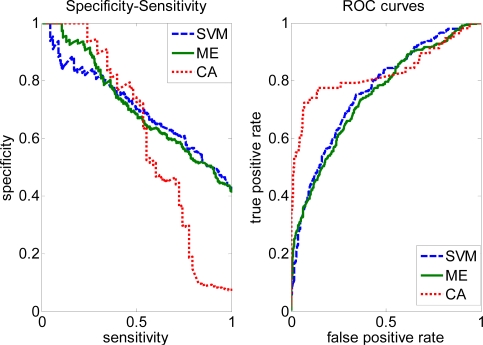
Specificity-sensitivity and ROC curves on the Trim data based on SVM, ME and CA.

**Table 1.** Performances on a dataset of 61 protein chains using 5-fold cross-validation.

**Dataset**	**Method**	**Sensitivity**	**Specificity**	**Accuracy**	**F1-mesure**	**CC**
Complete	CA	0.5612	0.5883	0.6962	0.5916	0.2893
	Random	0.4535	0.4764	0.5582	0.4462	0.0604
Trim	CA	0.6559	0.5334	0.6086	0.5863	0.2124
	Random	0.5036	0.4555	0.4955	0.4550	−0.0065

**Table 2.** Performances of SVM, CA and ME based on 5-fold cross-validation.

**Data set**	**Method**	**Sensitivity**	**Specificity**	**F1-measure**	**Accuracy**	**CC**
	SVM	0.5547	0.6294	0.5796	0.6896	0.2443
Complete	ME	0.5011	0.6734	0.5408	0.6761	0.2719
	CA	0.5612	0.5883	0.5916	0.6962	0.2893
	SVM	0.5807	0.5883	0.5639	0.6662	0.2032
Trim	ME	0.6103	0.6101	0.6576	0.5860	0.2417
	CA	0.6559	0.5334	0.5863	0.6086	0.2124

**Table 3.** Two types of data sets.

**Data set**	**Chains**	**Residues**	**Surface Residues**	**Interface residues**
Complete^a^	61	10,256	6,567	2,465
Trim^b^	61	10,256	2,465	2,465

a Include all surface residues;

b Remove randomly non-interface residues in order to equal with interface residues.
